# The efficacy and safety of short-term and low-dose IL-2 combined with tocilizumab to treat rheumatoid arthritis

**DOI:** 10.3389/fimmu.2024.1359041

**Published:** 2024-04-22

**Authors:** Sheng-Xiao Zhang, Hao-Ran Chen, Jia Wang, Hong-Fang Shao, Ting Cheng, Ruo-Meng Pei, Qin-Yi Su, He-Yi Zhang, Xiao-Feng Li

**Affiliations:** ^1^ Department of Rheumatology, the Second Hospital of Shanxi Medical University, Taiyuan, Shanxi, China; ^2^ Key Laboratory of Cellular Physiology at Shanxi Medical University, Ministry of Education, Taiyuan, Shanxi, China; ^3^ Shanxi Provincial Key Laboratory of Rheumatism Immune Microecology, The Shanxi Medical University, Taiyuan, Shanxi, China; ^4^ School of Management, Shanxi Medical University, Taiyuan, Shanxi, China; ^5^ Academy of Medical Sciences, Shanxi Medical University, Taiyuan, Shanxi, China; ^6^ School of Basic Medical Sciences, Shanxi Medical University, Taiyuan, China

**Keywords:** rheumatoid arthritis, low-dose IL-2, tocilizumab, immunoregulation, therapeutics

## Abstract

**Background:**

Immunotherapy targeting factors related to immune imbalance has been widely employed for RA treatment. This study aimed to evaluate the efficacy and safety of low-dose interleukin (IL)-2 combined with tocilizumab (TCZ), a biologics targeting IL-6, in RA patients.

**Methods:**

Fifty adults with active RA who met the criteria with complete clinical data were recruited, and divided into three groups: control group (n=15), IL-2 group (n=26), and IL-2+TCZ group (n=9). In addition to basic treatment, participants in the IL-2 group received IL-2 (0.5 MIU/day), while participants in the IL-2+TCZ group received IL-2 (0.5 MIU/day) along with one dose of TCZ (8 mg/kg, maximum dose: 800 mg). All subjects underwent condition assessment, laboratory indicators and safety indicators detection, and records before treatment and one week after treatment.

**Results:**

Compared with the baseline, all three groups showed significant improvement in disease conditions, as evidenced by significantly reduced disease activity indicators. The low-dose IL-2 and combination treatment groups demonstrated a violent proliferation of Tregs, while the absolute number of Th1, Th2, and Th17 cells in the latter group showed a decreasing trend. The decrease in the Th17/Treg ratio was more pronounced in the IL-2+TCZ groups. No significant adverse reactions were observed in any of the patients.

**Conclusion:**

Exogenous low doses of IL-2 combined TCZ were found to be safe and effective in reducing effector T cells and appropriately increasing Treg levels in RA patients with high effector T cell levels. This approach helps regulate immune homeostasis and contributes to the prevention of disease deterioration.

**Clinical trial registration:**

https://www.chictr.org.cn/showprojEN.html?proj=13909, identifier ChiCTR-INR-16009546.

## Introduction

Rheumatoid arthritis (RA) is a chronic autoimmune disease characterized by persistent and progressive inflammation of the synovial joints, leading to debilitating arthritis and heterogeneous extra-articular manifestations (1). The high incidence, teratogenicity, disability, and poor prognosis associated with RA present significant challenges and demand effective management strategies (2, 3). Currently, the clinical treatment of RA relies heavily on glucocorticoids, non-steroidal anti-inflammatory drugs (NSAIDs), and disease-modifying anti-rheumatic drugs (DMARDs) such as synthetic or biologic agents (4, 5). While these medications play a crucial role in alleviating joint symptoms and halting disease progression, they often come with potential side effects including nausea, anorexia, and bone marrow suppression due to individual variations (4, 5). As the need for new anti-RA therapies becomes increasingly urgent, both clinical and basic research efforts are continuously being conducted to better understand the pathogenesis of this complex disease.

The etiology of RA remains incompletely understood, with genetic, autoimmune, and environmental factors being considered as the primary causes (6). Individuals with genetic predisposition may experience abnormal immune responses in synovium triggered by environmental factors, resulting in synovitis characterized by joint swelling, pain, and irreversible damage (7). T lymphocyte subgroups and their released pro-inflammatory or anti-inflammatory cytokines exist widely in synovitis and play an indispensable role in perpetuating the inflammatory response (8, 9). Good progress has been made in the development and application of biological agents targeting proinflammatory cytokines, such as tumor necrosis factor-alpha (TNF-α), Interleukin (IL)-6, and IL-17 (10). IL-6, a pleiotropic pro-inflammatory cytokine with various biological activities, participates in a variety of biological activities such as immune regulation, hematopoiesis, and inflammatory response (11). IL-6 amplifies the effects of cytokines such as TNF-α and IL-17, indirectly influencing the activation of tyrosine protein kinases 1 and 2 via binding with IL-6R. This ultimately leads to the release of TNF-α, IL-17, and other cytokines, contributing to the development of arthritis and extra-articular lesions (12, 13). In addition, IL-6 promotes the proliferation, differentiation, and maturation of B cells, and subsequently causes serious antibody deposition in the synovium and exacerbation of joint injury (14). Up-regulation of IL-6 has even been observed prior to the onset of RA (15). As expected, the humanized anti-interleukin-6 receptor (IL-6R) antibody, tocilizumab (TCZ), has shown high efficacy in treating patients with RA. However, limitations still exist (16–18).

Evidence from numerous studies indicates that the imbalance of CD4+T cell subsets, especially the significant reduction in T regulatory cells (Tregs) responsible for maintaining immune homeostasis and suppressing inflammatory response, contributes to joint damage and disease deterioration in RA (19). Tregs have emerged as potential therapeutic targets for many autoimmune diseases such as RA (20, 21). Initial data indicate that the number of circulating Tregs in RA patients decreases with increasing disease activity, which can be reversed by exogenous supplementation with low doses of IL-2 (22). IL-2, a cytokine with multiple immunomodulatory functions, has been widely recognized for its ability to selectively expand Tregs and maintain or restore the balance of CD4+T subgroup balance and immune tolerance (23, 24). Encouraging progress has also been made in combining exogenous IL-2 with DMARDs such as methotrexate and TNF inhibitors, but not with TCZ (25, 26).

Previous studies have confirmed the efficacy and safety of low-dose IL-2 in the treatment of RA. In the current study, we analyzed the effects of low-dose IL-2 combined with TCZ on clinical symptoms, immune-related indicators, and CD4+T lymphocyte subsets in RA patients, and evaluated its safety.

## Methods

### Participants

A total of 50 adult RA patients with active disease [Disease Activity Score-28 with erythrocyte sedimentation rate (DAS28-ESR) score >3.2] were recruited for this study. Participants were screened according to the EULAR/American College of Rheumatology 2010 criteria and were admitted to the Rheumatology and Immunology Department of the Second Hospital of Shanxi Medical University from March 2017 to December 2019 (27). Exclusion criteria included the presence of other autoimmune diseases such as Sjogren’s syndrome, chronic infections such as Hepatitis B Virus (HBV) or Epstein-Barr Virus (EBV) infections, active HIV, tuberculosis, malignancies, hematological diseases, and vital organ dysfunction. Participants who had received bDMARDs with different mechanisms of action (such as anti-TNFα biological agent, rituximab, or any JAK inhibitor) during the 12 weeks prior to screening or the half-life of their respective drugs were also excluded.

### Study design

We previously reported that combined with low-dose IL-2[0.5 Million International Units (MIU)/day of IL-2 for five consecutive days] on the basis of previous treatment can promote the proliferation of circulating Tregs in patients with RA, PsA, and pSS, and significantly improve the symptoms of patients (22, 28, 29). Therefore, eligible participants were randomly assigned to three age-sex matched groups: control group (n=15), IL-2 group (n=26), and IL-2+TCZ group (n=9). In addition to basic treatment, participants in the IL-2 group received IL-2 (0.5 MIU/day) and participants in the IL-2+TCZ group received IL-2 (0.5 MIU/day) along with one dose of TCZ (8 mg/kg, maximum dose: 800 mg). All subjects underwent condition assessment, laboratory indicators and safety indicators detection, and records before treatment and one week after treatment.

### Assessments

Epidemiological data, course of disease, medical history, and medication history of all patients before and after treatment were performed at scheduled visits. Completed the assessment of the patient’s condition, including tender joint count (TJC), swollen joint count (SJC), VAS score, C-reactive protein (CRP), and critical organ function such as blood routine examination, alanine aminotransferase (ALT), aspartic transaminase (AST), blood urea nitrogen (BUN), and serum creatinine (Scr).

In addition, for patients receiving IL-2 or TCZ treatment, the absolute count and percentage of CD4+T lymphocyte subsets should be evaluated after treatment. In this study, the total number of peripheral CD4+T cells was measured using freeze-dried particles with known quantities of fluorescent beads and BD Trucount™ tubes. Flow cytometry was used to calculate the percentage of Th1(CD4+IFN-γ+), Th2 (CD4+IL-4+), Th17 (CD4+IL-17+) and Tregs (CD4+CD25+Foxp3+) in at least 5000 to 10,000 cells, and then the absolute count of each subpopulation was calculated according to the following formula: the absolute number of CD4+T subsets = the percentage of each CD4+T subset × the absolute number of total CD4+T cells. All the reagents, instruments, and analysis software in this experiment were from BD Company in the United States.

### Safety

The incidence and severity of all adverse events such as rash and fever were recorded. Blood routine, liver and kidney function, and other safety evaluation indexes were detected before and after treatment.

### Statistical analysis

SPSS25.0 and GraphPad Prism 9.0 software were used for statistical analysis and drawing. Categorical variables are expressed in frequency (percentage) and compared with χ^2^ tests. Continuous variables conforming to the normal distribution were described as mean ± standard error (SE) and inter-group or intra-group comparisons were performed by One-Way ANOVA or T-test; otherwise, they were described as median (P25, P75), and non-parametric tests were performed for inter-group or intra-group comparisons. *P*<0.05 (two-tailed) was considered statistically significant.

## Results

### Epidemiologic features and baseline values of general data

Following screening based on the inclusion and exclusion criteria, 15 RA patients (12 females and 3 males) with an average age of 55.13 ± 17.88 years and an average DAS28 score of 5.5 ± 1.35 were included in the control group, 26 recruiters (15 females and 11 males) with an average age of 55.65 ± 11.3 years and an average DAS28 score of 5.31 ± 1.18 were included in the IL-2 group, and 9 subjects (8 females and 1 male) with an average age of 45.67 ± 12.33 years and an average DAS28 score of 5.68 ± 1.02 were included in the IL-2+TCZ group. There were no statistically significant differences in age (F=1.881, P=0.164), gender (χ^2^ = 4.37, P=0.113), or DAS28 score (F=0.337, P=0.716) among the three groups. Compared with the IL-2 group, the IL-2+TCZ group had a shorter course of disease, and compared with the control group, the white blood count and neutrophils count in this group decreased, and the difference was statistically significant(P<0.05). ALT level in the IL-2+TCZ group was increased within the normal range than that in the control group (P<0.05), which had no clinical significance. There was no significant difference in other general indicators including ESR, CRP, TJC, SJC, PLT, or lymphocyte count among the three groups (P>0.05) (**Table 1**).

**Table 1:** Baseline characteristics of all participants (Mean±SE).

	Control group	IL-2 group	IL-2+tocilizumab group
	N=15	N=26	N=9
Duration, months	82.6 ± 18.78	136.81 ± 27.08	50.78 ± 19.68^b^
General laboratory index			
ESR, mm/h	60.93 ± 8.41	53.92 ± 7.01	52.56 ± 11.69
CRP, mg/L	57.18 ± 20.23	33.15 ± 7.42	23.48 ± 8.5
TJC	10.33 ± 2.24	10.54 ± 1.72	11.89 ± 2.85
SJC	5.33 ± 1.55	4.15 ± 0.99	7.89 ± 2.6
WBC, ×10^9^/L	7.33 ± 0.49	6.7 ± 0.49	5.48 ± 0.5^a^
RBC, ×10^9^/L	4.27 ± 0.12	4.27 ± 0.09	4.28 ± 0.19
Hb, g/L	123.97 ± 4.58	133.81 ± 12.25	116.32 ± 3.12
PLT, ×10^9^/L	307.35 ± 26.38	287.19 ± 18.51	269.7 ± 26.84
Lymphocyte, ×10^9^/L	1.72 ± 0.15	1.5 ± 0.1	1.61 ± 0.14
Neutrophil, ×10^9^/L	4.97 ± 0.46	4.62 ± 0.42	3.35 ± 0.37^a^
ALT, U/L	16.75 ± 1.75	17.26 ± 2.16	25.42 ± 4.17^a^
AST, U/L	18.27 ± 1.55	18.23 ± 1.45	25 ± 4.86
BUN, mmol/L	5.28 ± 0.36	4.88 ± 0.3	4.43 ± 0.59
CR, mmol/L	53.93 ± 4.57	54.32 ± 3.1	50.56 ± 6.82
Immune-related laboratory indicators			
C3, g/L	1.09 ± 0.09	1 ± 0.1	393.23 ± 192.97
C4, g/L	0.22 ± 0.03	0.24 ± 0.04	31.13 ± 8.23^ab^
RF, U/mL	268.44 ± 97.47	449.09 ± 117.72	23.33 ± 8.66^ab^
anti-CCP, RU/mL	384.57 ± 75	560.28 ± 118.93	691.85 ± 238.81
Anti-MCV, U/mL	369.85 ± 105.21	432.08 ± 75.36	564.64 ± 160.02
GPI	2.55 ± 0.55	0.46 ± 0.23	1.77 ± 0.43

^a^,^b^ indicated P<0.05 compared with the control group and IL-2 group, respectively.

### Baseline levels of immune-related laboratory indicators

We examined and analyzed baseline levels of immune-related markers, including the absolute numbers of CD4+T subsets, in three groups. Our results showed that complement (C)4 levels were significantly increased and RF levels decreased in the IL-2+TCZ group compared to both the IL-2 and control groups (P<0.05). Compared with the control group, the level of glucose-6-phosphate isomerase (GPI) in the IL-2 treatment group was significantly lower (P<0.05). Levels of C3, anti-cyclic citrullinated peptide (CCP) antibody, or anti-mutated citrullinated vimentin (MCV) antibody were similar among the three groups. (**Table 1**)

**Figure 1:**
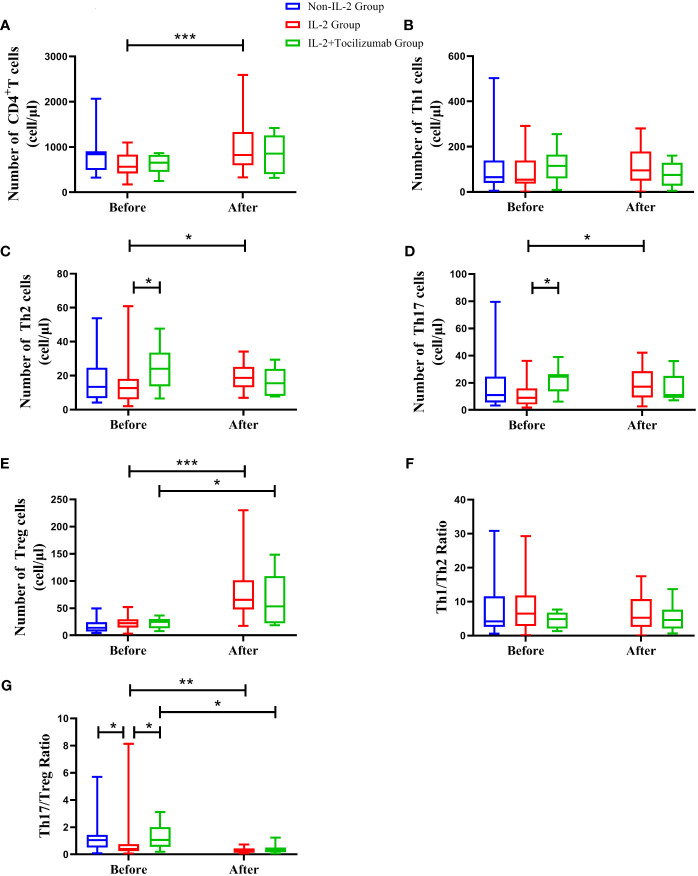
The absolute numbers of peripheral CD4+T lymphocytes **(A)** and its subsets such as Th1 **(B)**, Th2 **(C)**, Th17 **(D)**, and Treg cells **(E)** in the control group, IL-2 group, and IL-2+tocilizumab group during the observation period. The levels of Th1/Th2 ratio **(F)** and Th17/Treg ratio **(G)** in the above groups were shown. *P < 0.05, **P <0.01, ***P <0.001. The significance level is P<0.05.

Although the absolute number of CD4+T cells was similar among the three groups before treatment, there were still some differences among the subsets. The Th17/Treg ratio was lower in the IL-2 group compared to the other patients (P<0.05). What is most noteworthy is that the number of Th2 and Th17 in the IL-2+TCZ group was 24.05(13.66, 33.57) and 24.56 (13.72, 26.44) cells/µl, respectively, which was significantly higher than 12.61(6.13, 18.11) and 8.97 (4.13, 15.96) cells/µl in the IL-2 group, and the difference was statistically significant (P<0.05) (**Figure 1**).

### Disease activity levels before and after treatment

**Figure 2:**
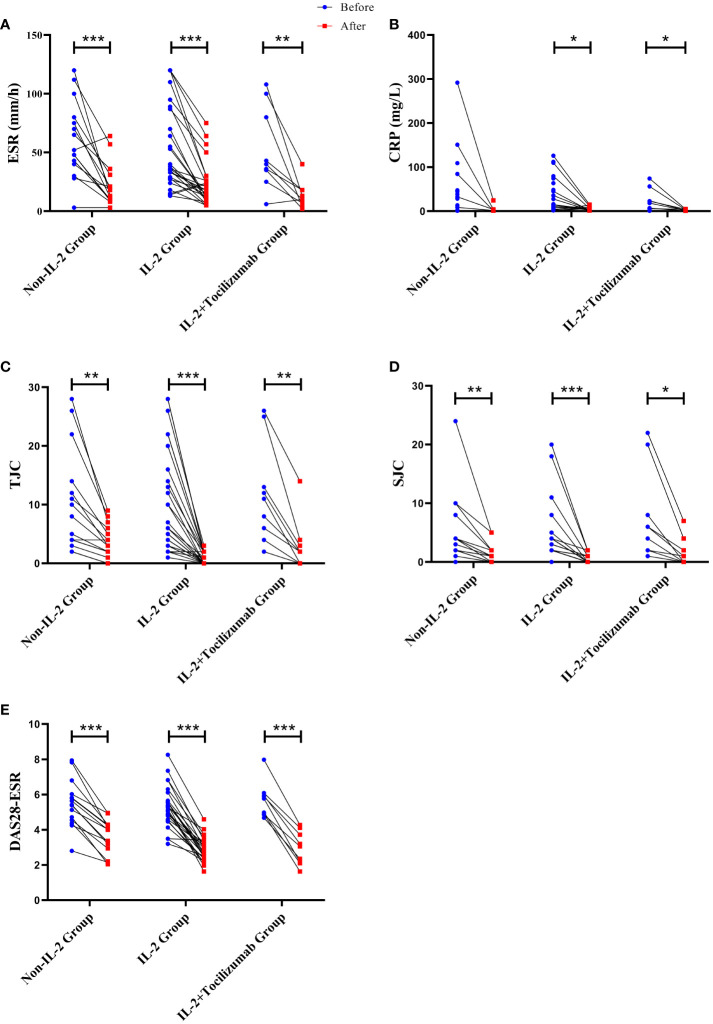
Changes of ESR **(A)**, CRP **(B)**, TJC **(C)**, SJC **(D)**, and DAS28-ESR **(E)** levels in three groups before and after treatment. *P < 0.05, **P <0.01, ***P <0.001. The significance level is P<0.05. ESR, erythrocyte sedimentation rate; CRP, C-reactive protein; TJC, tender joint counts; SJC, swollen joint counts; DAS28, disease activity score-28.

Compared with the baseline, the disease conditions of all three groups were significantly improved, and the indicators related to disease activity including DAS28, ESR, TJC, and SJC were significantly and meaningfully reduced, only the CRP levels of IL-2 and IL-2+TCZ group were significantly decreased (P<0.05). After treatment, the number of peripheral lymphocytes and neutrophils increased in all patients (P<0.05). Otherwise, neutrophil count was the highest in the control group, the lowest DAS28 score was in the IL-2 group, while the lowest CRP was in the IL-2+TCZ group (P<0.05). (**Figure 2**)

### The absolute counts of CD4+T subpopulations before and after treatment

In addition to improvements in arthritis, we evaluated CD4+T lymphocyte levels in patients treated with IL-2 and IL-2+TCZ after the corresponding regimen. Surprisingly, part of the CD4+T-lymphocyte subpopulation in both groups changed after a short period of intervention with a low dose of the medication. After low-dose IL-2 treatment, peripheral CD4+T [823(597, 1334) vs. 564(417, 832) cells/µl], Th2 [18.64(13.09, 25.20) vs. 12.61(6.13, 18.11) cells/µl], Th17 [17.23(9.32, 28.61) vs. 8.97(4.13, 15.96) cells/µl], and Treg cells [65.23(47.87, 101.58) vs. 22.59(14.35, 29.71) cells/µl] increased dramatically (P<0.05). On the contrary, the absolute number of Th1[74.58(27.31, 128.075) vs. 114.62(59.41, 165) cells/µl], Th2[15.47(8.13, 23.93) vs. 24.05(13.655, 33.565) cells/µl], and Th17 cells [11.04(8.89, 25.105) vs. 24.56(13.72, 26.44) cells/µl] in IL-2+TCZ group showed a decreasing trend without statistically significant (P>0.05). On day 7, the primary efficacy endpoint was reached with an increase of Tregs to a mean of 65.23 (47.87,101.575) cells/µl in the IL-2 group, equivalent to a three-fold increase (P<0.001); while Tregs increased to 53.17 (22.295,108.98) cells/µl on average in the IL-2+TCZ group, equivalent to a two-fold increase (P=0.021), but there was no significant difference between the two groups after treatment. Although the Th17/Treg ratio decreased significantly in both IL-2 and IL-2+TCZ groups, the decrease in the latter group was more amplified (P<0.05). (**Figure 1**; **Supplementary Figure 1**)

### Analysis of the correlation between the CD4+T subgroup and disease activity

**Figure 3:**
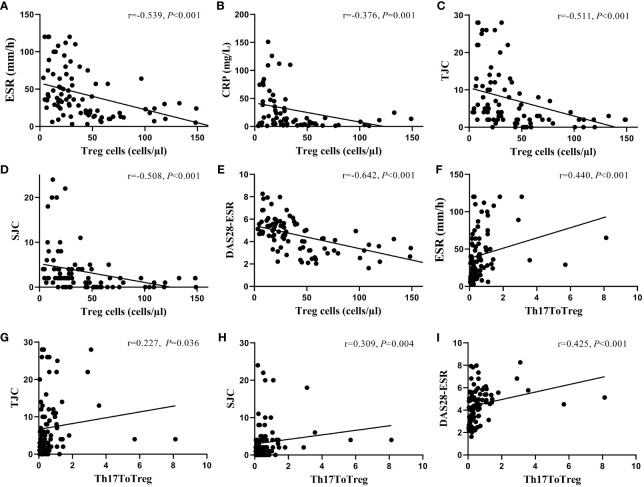
The correlation between the absolute count of circulating Treg cells and the ratio of Th17 to Treg cells and disease activity. The absolute count of Treg cells was negatively correlated with ESR **(A)**, CRP **(B)**, TJC **(C)**, SJC **(D)**, and DAS28-ESR levels **(E)**, while the ratio of Th17 to Treg cells was positively correlated with ESR **(F)**, TJC **(G)**, SJC **(H)**, and DAS28-ESR levels **(I)**. ESR, erythrocyte sedimentation rate; CRP, C-reactive protein; TJC, tender joint counts; SJC, swollen joint counts; DAS28, disease activity score-28; Treg cells, regulatory T cells.

We further evaluated CD4+T-lymphocyte subgroups that were significantly associated with measures of patient disease activity by Spearman correlation analysis. The results showed that the absolute count of Tregs and DAS28 (r=-0.642, P<0.001), ESR (r=-0.539, P<0.001), CRP(r=-0.376, P=0.001), TJC(r=-0.511, P<0.001), and SJC(r=-0.508, P<0.001) showed a significant negative correlation, while Th17/Treg was positively correlated with DAS28 (r=0.425, P<0.001), ESR (r=0.440, P<0.001), TJC(r=0.227, P=0.036), and SJC(r=0.309, P=0.004). (**Figure 3**)

### Safety

All patients did not have obvious adverse reactions such as allergy, fever, or infusion reaction, and could insist on completing treatment and efficacy evaluation. Despite ALT and BUN levels being slightly increased after IL-2 and TCZ treatment, they were all within the normal range and had no special clinical significance. (**Table 2**)

**Table 2:** Levels of general indicators in all patients after treatment(Mean±SE).

	Control group	IL-2 group	IL-2+tocilizumab group
DAS28	3.6 ± 0.25	2.85 ± 0.13^a^	2.95 ± 0.32
ESR, mm/h	22.07 ± 4.63	22.77 ± 3.66	13.56 ± 3.79
CRP, mg/L	7.06 ± 4.4	5.8 ± 1.07	2.66 ± 0.57
TJC	3.73 ± 0.72	0.96 ± 0.2^a^	3 ± 1.45
SJC	1.4 ± 0.42	0.42 ± 0.14^a^	1.67 ± 0.8
WBC, ×109/L	11.82 ± 1.08	8.55 ± 0.62^a^	7.15 ± 0.73^a^
RBC, ×109/L	4.14 ± 0.1	4.24 ± 0.11	4.48 ± 0.15
Hb, g/L	118.87 ± 4.34	124.65 ± 3.81	122.54 ± 2.88
PLT, ×109/L	273.33 ± 18.91	256.42 ± 16.57	230.22 ± 26.37
LY, ×109/L	2.57 ± 0.4	2.18 ± 0.18	2.4 ± 0.3
NEU, ×109/L	8.28 ± 0.95	5.76 ± 0.56^a^	4.15 ± 0.63^a^
ALT, U/L	28.91 ± 4.24	29.14 ± 4.45	22.34 ± 3.84
AST, U/L	17.65 ± 2.33	17.9 ± 1.98	15.46 ± 1.54
BUN, mmol/L	6.48 ± 0.45	5.79 ± 0.36	6.54 ± 1.09
CR, mmol/L	56.5 ± 2.08	60.05 ± 3.3	57.63 ± 7.82

^a^indicated P<0.05 compared with the Control group.

## Discussion

We have previously reported that Tregs are significantly reduced with the aggravation of disease in RA patients, and combined with low-dose IL-2 therapy can maintain or even rebuild autoimmune homeostasis by promoting the proliferation of Treg cells, thus delaying the deterioration of disease (22, 30). Great heterogeneity exists in the clinical presentation and immune mechanisms of RA, as a consequence, not all patients respond similarly to the same drug. This study aims to observe the effects of low-dose IL-2 combined with TCZ on arthritis symptoms and immune cells in active RA patients, based on the good results of low-dose IL-2 combined with bDMARDs, excluding TCZ, reported in previous studies (25, 26). The results of this study showed that low-dose IL-2 combined with TCZ treatment could safely and effectively reduce arthritis symptoms in RA patients, promote the more stable proliferation of Tregs, and reduce the proliferation of Th2, Th17, and Th17/Treg caused by IL-2 treatment alone. This is of great value for patients with immune disorders mediated by high levels of Th2 and Th17.

The dysregulation of the immune response, mainly including the imbalance of immune cells and cytokines, has been widely proven to contribute to the pathogenesis and progression of RA (31). More notably, the disorders of immune modulate cells not only exacerbate synovitis, progressive bone injury, and progressive destruction of articular cartilage but are also closely associated with complications such as pulmonary interstitial fibrosis and coronary artery disease that increase mortality in RA patients (32, 33). Therefore, targeting cytokines or immunomodulatory drugs are gradually entering clinical therapy.

According to the production of cytokines and the expression of main transcription factors, CD4+T cells are roughly divided into CD4+T helper (Th) cells such as Th1, Th2, and Th17 cells, which can regulate the inflammatory environment, promote antibody production, control innate immunity and stimulate immune memory, and anti-inflammatory CD4+T groups, namely Treg, which inhibit inflammation, dominant self-tolerance, maintain immune homeostasis and control immune responses to prevent autoimmune diseases (34). Inflammation is usually accompanied by dysregulation of CD4+T cell subsets and cytokine networks, which play a dominant role in immune regulation, characterized by excessive proliferation of inflammatory cells or depletion or dysfunction of regulatory cells, and ultimately the inflammation gets aggravated (19, 21). As observed in this study, the absolute count of peripheral Tregs and the ratio of Th17 to Treg were strongly associated with the disease activities of RA. Specifically, the absolute counts of Treg cells were significantly negatively correlated with the DAS28, ESR, CRP, TJC, and SJC levels, while Th17/Treg was significantly positively correlated with DAS28, ESR, TJC, and SJC, which demonstrated the regulation of Treg cells levels and Th17/Treg ratio were beneficial to the recovery of RA.

IL-2, produced by T-cell activation, is an essential cytokine for lymphocyte growth. T cell proliferation in mice with knockout of the IL-2 receptor and IL-2 expression gene was uncontrolled and combined with progressive fatal systemic autoimmune disease, which could be prevented by adoptive transfer of Tregs, highlighting the importance of effective IL-2 signaling in immune function (35, 36). As demonstrated in this study, low doses of IL-2 can improve the condition by promoting Tregs proliferation in RA patients. Consistent with the findings, a prospective study performed by Rosenzwajg M. demonstrated the value of low-dose IL-2 in 11 autoimmune diseases, proposing that all patients had relatively specific amplification and activation of Tregs, and were well tolerated to low-dose IL-2 regardless of disease and concomitant therapy (24). However, IL-2 is regarded as the central factor responsible for the proliferation, differentiation, and function of CD4+T cell subsets, and can also promote or inhibit the expression of specific Th cell gene programs, including Th2, and Th17 cells (37). This action of IL-2 signaling is extremely detrimental in patients with immune disorders characterized by high levels of Th cells such as Th2. Therefore, it may be feasible to co-regulate immune homeostasis with other DMARD drugs.

Given the complexity of the immune network in RA, biological agents targeting immune cells and cytokines have emerged in an endless stream in recent years, among which infliximab targeting TNF-α and TCZ targeting IL-6R are the most widely used in clinical practice. Circulating IL-6 levels are elevated in RA patients, and there is a correlation between IL-6 levels in the affected joints and inflammatory markers. IL-6 is required for the development of collagen-induced arthritis (CIA), and blocking IL-6 receptors improved joint disease in CIA mice (38, 39). IL-6R-mediated JAK-STAT signaling pathway induces or aggravates RA lesions by promoting B cell activation and T cell proliferation (12, 13). The binding of IL-6 to its receptor induces the expression of RORγt by phosphorylation of JAK-mediated STAT3, thereby promoting the release of pro-inflammatory cytokines IL-17 and IL-22, recruiting granulocytes, and promoting the development of an amplifying loop for inflammation (40).

Tocilizumab, the first humanized monoclonal antibody against IL-6R, could inhibit all IL-6R-mediated signaling by intravenous or subcutaneous injection, alone or in combination with methotrexate, for the treatment of RA in adults who have either responded inadequately to or were intolerant of previous therapy with ≥ 1 csDMARDs or TNF inhibitor (41). Our study observed that in RA patients with high levels of Th2, Th17, and Th17/Treg on the basis of the original treatment, exogenous TCZ and low dose of IL-2 can rapidly and safely reduce the DAS28 score and alleviate the disease in a short period, effectively promote the moderate proliferation of Tregs and reduce the level of Th cells, which is consistent with the previous results of TCZ combined with other DMARDs (42). Meanwhile, more female patients were recruited in this study, but relevant literature showed that gender did not affect the distribution ratio of peripheral blood immune cells and the therapeutic effect of tocilizumab, so it was believed that gender did not affect the judgment of results in this study (43).

Our study has a few limitations, such as a single-center study with a small sample size and short observation time. Additionally, the lack of cytokine data was considered a limitation of this study. Since this was a retrospective study, data from all patients were not available for several variables.

In conclusion, this study further confirms that reduced Tregs are an integral factor in the deterioration of RA, which can be improved or even reversed by exogenous low doses of IL-2. However, combined TCZ can safely and effectively reduce Th cells while appropriately Treg for RA patients with high Th cell levels, thus regulating immune homeostasis and contributing to preventing disease deterioration.

## Data availability statement

The original contributions presented in the study are included in the article/**Supplementary Material**. Further inquiries can be directed to the corresponding author.

## Ethics statement

The studies involving humans were approved by This study was approved by the Ethics Committee of the Second Hospital of Shanxi Medical University (2016 KY-007). The studies were conducted in accordance with the local legislation and institutional requirements. The participants provided their written informed consent to participate in this study.

## Author contributions

SZ: Investigation, Methodology, Resources, Writing – original draft. HC: Data curation, Software, Writing – original draft. JW: Investigation, Methodology, Resources, Writing – original draft. HS: Formal analysis, Methodology, Supervision, Writing – review & editing. TC: Data curation, Investigation, Writing – original draft. RP: Data curation, Methodology, Validation, Writing – review & editing. QS: Methodology, Validation, Writing – original draft. HZ: Investigation, Methodology, Software, Writing – review & editing. XL: Conceptualization, Resources, Writing – review & editing.
