# Supplementary material for: The efficacy and safety of short-term and low-dose IL-2 combined with tocilizumab to treat rheumatoid arthritis
Source: Front Immunol. 2024 Apr 22;15:1359041. doi: 10.3389/fimmu.2024.1359041 (PMC11070481; doi:10.3389/fimmu.2024.1359041)
Supplement: Supplementary file 1 [file DataSheet_1.docx]

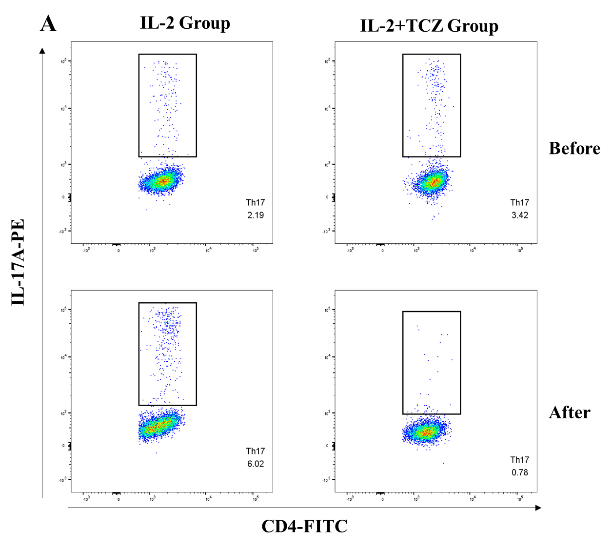

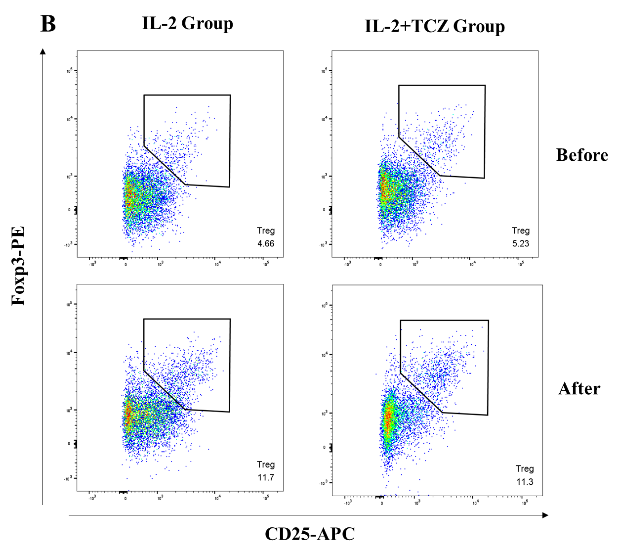


Supplementary figure 1：Representative flow cytometry showed significant changes in the levels of Th17 and Treg cells after low-dose IL-2 or IL-2+TCZ treatment, respectively.
